# Amino-terminal extension present in the methionine aminopeptidase type 1c of *Mycobacterium tuberculosis *is indispensible for its activity

**DOI:** 10.1186/1471-2091-12-35

**Published:** 2011-07-05

**Authors:** Pavitra Kanudia, Monica Mittal, Sangaralingam Kumaran, Pradip K Chakraborti

**Affiliations:** 1 Institute of Microbial Technology, Council of Scientific and Industrial Research, Sector 39A, Chandigarh 160 036, India

## Abstract

**Background:**

Methionine aminopeptidase (MetAP) is a ubiquitous enzyme in both prokaryotes and eukaryotes, which catalyzes co-translational removal of N-terminal methionine from elongating polypeptide chains during protein synthesis. It specifically removes the terminal methionine in all organisms, if the penultimate residue is non-bulky and uncharged. The MetAP action for exclusion of N-terminal methionine is mandatory in 50-70% of nascent proteins. Such an activity is required for proper sub cellular localization, additional processing and eventually for the degradation of proteins.

**Results:**

We cloned genes encoding two such metalloproteases (*Mt*MetAP1a and *Mt*MetAP1c) present in *Mycobacterium tuberculosis *and expressed them as histidine-tagged proteins in *Escherichia coli*. Although they have different substrate preferences, for Met-Ala-Ser, we found, *Mt*MetAP1c had significantly high enzyme turnover rate as opposed to *Mt*MetAP1a. Circular dichroism spectroscopic studies as well as monitoring of enzyme activity indicated high temperature stability (up to 50°C) of *Mt*MetAP1a compared to that of the *Mt*MetAP1c. Modelling of *Mt*MetAP1a based on *Mt*MetAP1c crystal structure revealed the distinct spatial arrangements of identical active site amino acid residues and their mutations affected the enzymatic activities of both the proteins. Strikingly, we observed that 40 amino acid long N-terminal extension of *Mt*MetAP1c, compared to its other family members, contributes towards the activity and stability of this enzyme, which has never been reported for any methionine aminopeptidase. Furthermore, mutational analysis revealed that Val-18 and Pro-19 of *Mt*MetAP1c are crucial for its enzymatic activity. Consistent with this observation, molecular dynamic simulation studies of wild-type and these variants strongly suggest their involvement in maintaining active site conformation of *Mt*MetAP1c.

**Conclusion:**

Our findings unequivocally emphasized that N-terminal extension of *Mt*MetAP1c contributes towards the functionality of the enzyme presumably by regulating active site residues through "action-at-a-distance" mechanism and we for the first time are reporting this unique function of the enzyme.

## Background

The N-terminal methionine excision (NME) is an essential co-translational proteolytic process responsible for the diversity of amino-termini of proteins in both prokaryotes and eukaryotes [1]. It is an irreversible reaction, which occurs soon after N-terminal residues of the nascent polypeptide chain emerge from the ribosome exit tunnel before the commencement of protein folding [2]. The enzyme involved in the process is known as methionine aminopeptidase (MetAP), which specifically removes the terminal methionine in all organisms, if the penultimate residue is non-bulky and uncharged [3]. In prokaryotes and eukaryotic organelles where the initiator methionine is formylated, NME requires the action of another metalloprotease, peptide deformylase in addition to MetAP, which removes the N-formyl group present on all synthesized nascent polypeptides. In fact, removal of N-formyl group is a prerequisite for the subsequent action of MetAP [4]. Nevertheless, the MetAP action for exclusion of N-terminal methionine is mandatory in 50-70% of nascent proteins. Such an activity is required for proper sub cellular localization, additional processing and eventually for the degradation of proteins [5].

MetAPs are ubiquitous in distribution and are highly conserved throughout the phylogeny. They were initially grouped in two classes, MetAP1 and MetAP2 on the basis of sequence comparison. The type 2 enzymes, in contrast to type 1, have an approximately 60 amino acid long α helical domain inserted within the catalytic region of the enzyme [6,7]. This helical sub-domain shares neither sequence nor structural homology with any other known protein. The MetAPs are further divided in two subclasses on the basis of absence (subclass a) or presence (subclass b) of N-terminal extension [8]. The N-terminal extension present in type 1b contains two zinc finger motifs while that of type 2b has alternate stretches of polyacidic and polybasic residues. Recently, two new subclasses, type 1c and 2c, have been introduced where ~40 amino acid long N-terminal extension is present but zinc finger motif is absent [9,10].

Apart from several types of MetAPs, presence of more than one copy of functional genes encoding this enzyme has been reported in both prokaryotes and eukaryotes. In human, there are two such well characterized enzymes, which have been shown to be involved in cell proliferation [11,12]. Among four isoforms of MetAPs in *Plasmodium falciparum*, three exhibited enzymatic activities [13]. Genetic studies further established the essentiality of this enzyme in both prokaryotes and eukaryotes [14-16]. These findings led to the assumption that MetAP would be an ideal drug target and screening/designing of inhibitors against this enzyme might help in planning to combat the problem of rapid resurgence of diseases due to emergence of drug resistant strains of different pathogens. In this context, we focussed on tuberculosis, which is caused by *Mycobacterium tuberculosis*, and responsible for considerable human mortality throughout the world in recent years.

Although bacteria generally have single MetAP1, mycobacterial genome revealed the presence of 2-4 putative genes for MetAP1; for instance *M. tuberculosis *has two genes *mapA *(Rv0734) and *mapB *(Rv2861c) encoding *Mt*MetAP1a and *Mt*MetAP1c respectively. Although, crystal structure of *Mt*MetAP1c was solved as an apoenzyme and in complex form [9], biochemical characterization of the two *Mt*MetAPs has not been reported until very recently [17-21]. However, detailed structure-function analysis of these enzymes has not yet been carried out. In this scenario, we concentrated on both these *Mt*MetAP1s and in this article we report their distinct characteristics. Furthermore, we provide here evidence that catalytic domain of *Mt*MetAP1c alone is not sufficient for the enzyme activity.

## Results

### *Mt*MetAP1a and *Mt*MetAP1c exhibit subtle difference in their behaviour

**Figure 1:**
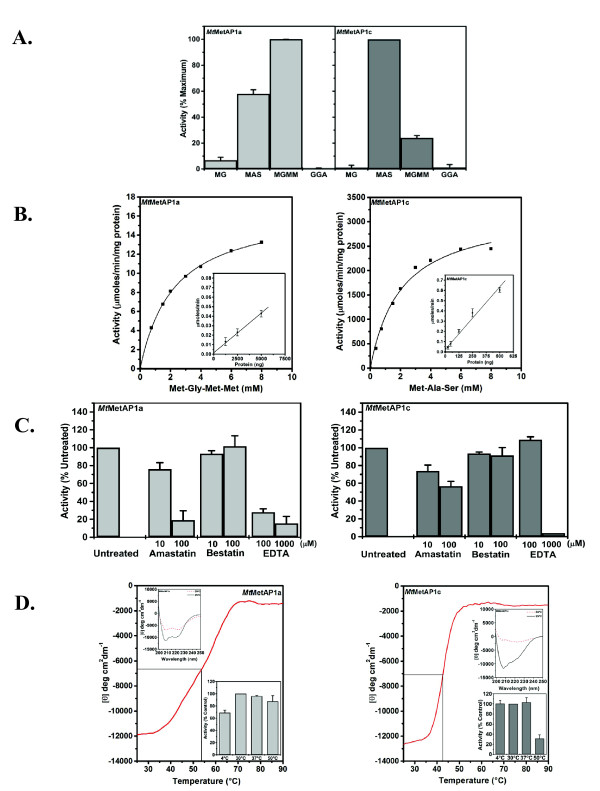
***Mt*MetAP1a and *Mt*MetAP1c are enzymatically active**. (A) Methionine aminopeptidase activity with different substrates. Enzyme activity of the two methionine aminopeptidases was determined using different substrates (4 mM) as indicated and *Mt*MetAP1a (1.7 nM) or *Mt*MetAP1c (0.038 nM) of purified protein as mentioned in the methods. Notations used: MG, Met-Gly; MAS, Met-Ala-Ser; MGMM, Met-Gly-Met-Met; GGA, Gly-Gly-Ala. (B) Kinetic analysis of methionine aminopeptidase activity of *Mt*MetAP1s. Kinetic analysis of methionine removal ability of *Mt*MetAP1a (left) was carried out by using 1.7 nM of purified protein with increasing concentration of Met-Gly-Met-Met as substrate. The reaction was monitored for 15 min. 0.038 nM of purified protein was incubated for 5 min with increasing concentration of Met-Ala-Ser as substrate to determine the kinetic parameters of *Mt*MetAP1c (Right). *Insets*, effect of increasing amount of protein on the enzyme activity. (C) Effect of different inhibitors and chelator on *Mt*MetAPs activity. MetAP1s were preincubated for 15 min at room temperature with the indicated amount of the inhibitors/EDTA and then the activity assay was performed. (D) Effect of temperature on *Mt*MetAP1s. The thermal unfolding was performed at the rate of 1°C/min and the change in the CD signal was monitored at 208 nm. *Left *and *right panels *represent the thermal denaturation graphs for *Mt*MetAP1a and *Mt*MetAP1c respectively. Upper insets show the absorption spectra of *Mt*MetAP1a and *Mt*MetAP1c monitored at 25°C and 50°C. *Lower insets *show the effect of preincubation for 10 min at indicated temperatures on the activity of two *Mt*MetAP1s. *Mt*MetAP1a and *Mt*MetAP1c are depicted at (left) and (right) respectively. The assay was carried out at 30°C after preincubation.

The *mapA *and *mapB *genes from *M. tuberculosis *were PCR amplified, cloned in pET-28c and expressed in *E. coli *strain BL21(DE3). The recombinant N-terminal His-tagged proteins were purified to near homogeneity by immobilized metal affinity chromatography using Ni-NTA resin. The purified proteins, on resolving in SDS-PAGE when visualized in Coomassie Brilliant Blue stained gels, exhibited bands at 37.3 ± 1.7 kDa (*n *= 4) and 39.1 ± 1.2 kDa (*n *= 4) for *Mt*MetAP1a and *Mt*MetAP1c, respectively. The authenticity of the expressed proteins was further confirmed by Western blotting using anti-His antibody (data not shown). *Mt*MetAP1a and *Mt*MetAP1c displayed maximum activity with Met-Gly-Met-Met and Met-Ala-Ser respectively. The enzymatic specificity for the terminal methionine residue was confirmed by using Gly-Gly-Ala as the substrate and as expected both the enzymes were unable to hydrolyze it (Figure 1A). Furthermore, using different peptides as substrates revealed that at least a tripeptide is the prerequisite for their functionality, since both the enzymes showed almost negligible activity with the dipeptide Met-Gly.

Activities of purified enzymes (1.7 nM for *Mt*MetAP1a or 0.038 nM for *Mt*MetAP1c) as a function of increasing concentrations (0.75-8 mM) of substrate (Met-Gly-Met-Met for *Mt*MetAP1a or Met-Ala-Ser for *Mt*MetAP1c) yielded typical Michaelis-Menten curves (Figure 1B). Interestingly, kinetic parameters determined by fitting the data to Michaelis-Menten equation for both the enzymes for their preferred substrate indicated that they were active (Table 1; see *K*_m _and *k*_cat _values with Met-Gly-Met-Met for *Mt*MetAP1a and Met-Ala-Ser for *Mt*MetAP1c; considering recombinant His-tagged proteins). Interestingly, using Met-Ala-Ser as the substrate for both the enzymes the catalytic efficiency of *Mt*MetAP1c was strikingly high (~350-fold) compared to that of the *Mt*MetAP1a (Table 1; *k*_cat_/*K*_m _= ~3 mM^-1^s^-1 ^as opposed to ~1156 mM^-1^s^-1^). Thus, our result is in consonance with a recent report that *Mt*MetAP1c is catalytically more efficient compared to *Mt*MetAP1a [20]. Expectedly, both the enzymes exhibited a linear increase in activity as a function of increasing protein concentrations (r = 0.997 or 0.991; Figure 1B, *insets *at both *left *and *right panels*).

**Table 1:** Kinetic parameters of *Mt*MetAPs

System	***K***_**m **_**(mM)**	***k***_**cat **_**(s**^**-1**^**)**	***k***_**cat**_**/*K***_**m **_**(mM**^**-1 **^**s**^**-1**^**)**	No. of experiments
***Mt*MetAP1a (MGMM)**	2.85 ± 0.69	9.63 ± 1.42	3.47 ± 0.7	3

***Mt*MetAP1a (MAS)**	3.3 ± 0.35	11.17 ± 0.97	3.32 ± 0.18	4

***Mt*MetAP1c (MAS)**	1.36 ± 0.25	1516 ± 174	1156 ± 317	4

Enzyme assays were carried out as described under 'Methods' following the incubation of purified *Mt*MetAP1a (1.7 nM/reaction) or *Mt*MetAP1c (0.038 nM/reaction) proteins with different concentrations (0.75-8 mM) of Met-Ala-Ser (MAS) or Met-Gly-Met-Met (MGMM) as substrate. *K*_m _and *V*_max _values were calculated from Non-linear regression analysis by fitting the data to Michaelis-Menten equation, and the results are expressed as mean ± SD. For calculating *k*_cat _values, the molecular mass of recombinant enzyme(s) was considered as 29.5 kDa (for *Mt*MetAP1a) or 33.1 kDa (for *Mt*MetAP1c). Mean ± SD for *k*_cat_/*K*_m _was determined after calculating it for individual experiment.

Since MetAPs are known as dinuclear metalloproteases, we also monitored the mycobacterial enzyme activities in response to EDTA, a metal ion chelator. As expected, metal ion chelator affected the activities of both the enzymes. However, compared to that used for *Mt*MetAP1c enzyme activity of *Mt*MetAP1a was inhibited at a lower concentrations of EDTA (Figure 1C). Leucine aminopeptidase inhibitors like amastatin and bestatin, have already been shown to inhibit MetAP activity by binding to its metal centre [7]. As shown in Figure 1C, amastatin affected both *Mt*MetAP1a and *Mt*MetAP1c enzyme activities; magnitude of inhibition, however, varied considerably between them (compared to untreated control, ~80% decrease in *Mt*MetAP1a as opposed to ~45% in *Mt*MetAP1c with 100 μM of amastatin). Bestatin, on the other hand, had no effect on the enzymatic activities of both the proteins within the concentration range tested in our experimental conditions.

We further addressed the adaptability of *Mt*MetAP1a and *Mt*MetAP1c enzymes to different temperatures. For this purpose, both the enzymes following incubation at different temperatures (4° or 30° or 37° or 50°C for 10 min) were assayed at 30°C for their activities. The maximum activity for both the enzymes was noticed between 30°-37°C. Interestingly, *Mt*MetAP1a retained ~85% of its activity at 50°C as opposed to ~25% by the *Mt*MetAP1c compared to that observed at 30°C (insets showing enzyme activities in left and right panels of Figure 1D). This observation led to the postulation that alteration in the enzyme activity might be occurring as a result of heating and this would very likely cause structural changes in both the proteins. This possibility was explored by incubating both the proteins to 50°C and comparing their far-UV CD spectra between 190-250 nm. *Mt*MetAP1a retained (> 50%) of its secondary structure on heating at 50°C while *Mt*MetAP1c was unfolded (compare insets showing CD spectrum in left and right panels of Figure 1D). In fact, determination of T_m _of both the proteins by CD spectroscopy yielded values of 53.6°C for *Mt*MetAP1a and 42.7°C for *Mt*MetAP1c (Figure 1D). These findings, therefore, established the difference in the adaptive ability of *Mt*MetAP1a to high temperature compared to that of the *Mt*MetAP1c and bring an end to the argument about the temperature tolerance of the *Mt*MetAP1s [17,20]. Thus, all these lines of evidence implicated the existence of subtle differences in the behaviour of two mycobacterial MetAPs.

### Contribution of active site residues on *Mt*MetAP1 enzyme activity

**Figure 2:**
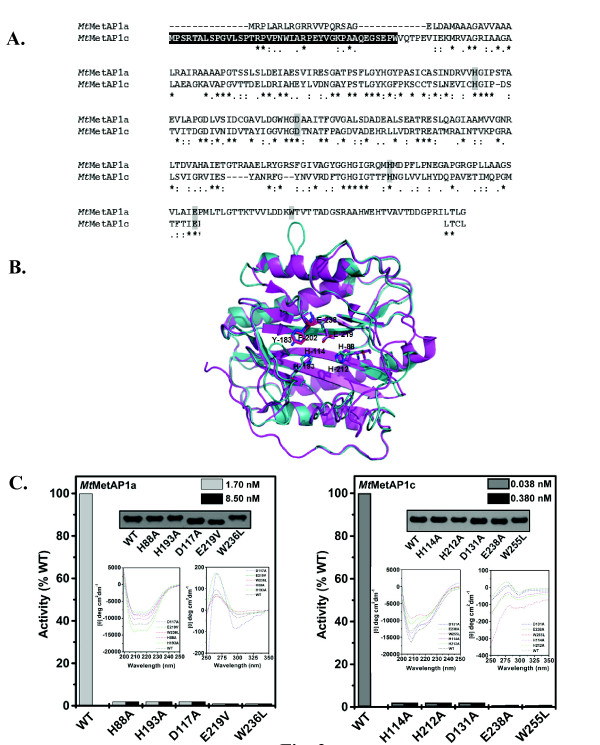
**Mutations of active site residues affect enzymatic activity of *Mt*MetAPs**. (A) Sequence alignment of the two mycobacterial methionine aminopeptidases was performed using Clustal X. Gaps in the sequences were introduced for optimum alignment. Asterisk and dots denote, identical and similar amino acids, respectively. Residues highlighted with black represent the 40 amino acid long N-terminal extension present in *Mt*MetAP1c and those with gray are the mutated amino acids in the two *Mt*MetAPs. (B) Structural alignment of *Mt*MetAP1a (green) with respect to *Mt*MetAP1c (pink). Residues in green (sticks) represent *Mt*MetAP1a active site residues (His-88, His-193, Glu-219) and residues in pink (sticks) depict *Mt*MetAP1c active site residues (His212, His-114, Glu-238). Residue in blue is Tyr-183 (*Mt*MetAP1a) and in hot pink is Phe-202 (*Mt*MetAP1c). (C) Methionine aminopeptidase activity of different point mutants. Enzyme activity for the indicated amount of wild-type and purified variants of two *Mt*MetAP1s was monitored using 4 mM of substrate, Met-Gly-Met-Met (*Mt*MetAP1a) and Met-Ala-Ser (*Mt*MetAP1c). *Insets*, Western blot of mutant proteins using anti-His antibody, far-UV and near-UV CD spectra.

Nucleotide derived amino acid sequence alignment of *Mt*MetAP1a with *Mt*MetAP1c revealed ~41% identity (Figure 2A) and the crystal structure of *Mt*MetAP1c (PDB ID: 1YJ3) has already been resolved. Since *Mt*MetAP1a did not show any significant identity with any other protein in the PDB database, we used *Mt*MetAP1c as the template for modelling the structure of this metalloprotease [9]. The modelled structure of *Mt*MetAP1a was validated by PROCHECK and it shows that 98% residues are in the most favoured regions of Ramachandran plot. The monomer of each protein consists of two pairs of α-helices (α1 to α4) packed on the either side of the central antiparallel β-sheet (β3 and β4). Interestingly, ~55% of the structure comprised of loops connecting the secondary structural elements. However, the 40 amino acid long N-terminal extension of *Mt*MetAP1c, which is devoid of any secondary structural element, lies across the surface of its catalytic domain (Figure 2B).

Structural analysis predicted the presence of His-88, His-193 and Glu-219 in the active site of *Mt*MetAP1a, while residues like Asp-106, Asp-117, Glu-250 and Trp-236 are lining the perimeter of the active site. The presence of homologous residues in *Mt*MetAP1c implies that the active site of both the proteins is essentially conserved (Figure 2A). To gain an insight on their contribution towards the enzyme activity, we generated several point mutants (H88A, H193A, D117A, E219V, W236L for *Mt*MetAP1a and H114A, H212A, D131A, E238A, W255L for *Mt*MetAP1c). Interestingly, the variants do not show any appreciable enzymatic activity even on using 10-fold excess protein compared to that of the wild-type (Figure 2C). In fact, mutating W236L in *Mt*MetAP1a and W255L in *Mt*MetAP1c led to complete loss in the activity (Figure 2C), which is in contrast to the report available with MetAP1 of *E. coli *[22] . Expression of all the variants, however, was authenticated by Western blotting using anti-His antibody (*insets*, Figure 2C). To examine the effect of mutations on the secondary and tertiary structures of the *Mt*MetAP1s, the CD spectral studies were performed. Far-UV CD spectrum of the mutant proteins of *Mt*MetAP1a revealed that H88A, D117A, E219V and W236L show reduction in helical content. The trend of the data in the region of 200-210 nm suggests that the band with negative mean residue ellipticity at lower wavelengths has reduced signal strength presumably owing to the conversion of some helical regions to less ordered structures (Figure 2C, *left panel, inset*). Interestingly, mutant H193A shows greater helicity than the wild-type protein. Far-UV CD spectral studies of wild-type *Mt*MetAP1c and its variants revealed that in D131A although there is no significant reduction in the mean residue ellipticity at the 200-210 nm range, it decreased at the 220-225 nm range (Figure 2C, *right panel, inset*). On the other hand, in H114A and E238A reduction in band intensity was observed at both 200-210 nm and 220-225 nm. In H212A and W255L the band intensities at the 200-210 nm as well as in 220-225 nm were further reduced (Figure 2C, *right panel, inset*). Thus, the mutations affected secondary structure of both the proteins and changes observed in the near-UV CD spectra indicated alteration in their aromatic environment (*insets *of Figure 2C, see both *left *and *right panels*).

### Role of N-terminal extension of *Mt*MetAP1c on the enzyme activity

**Figure 3:**
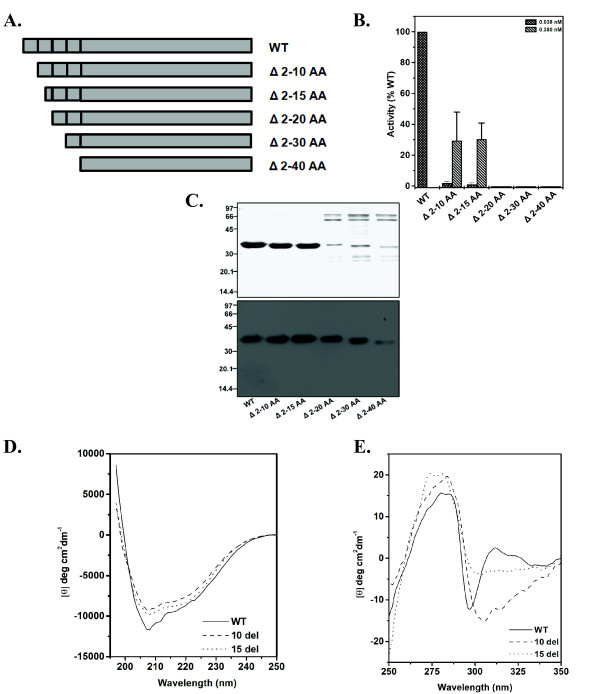
**N-terminal deletion of *Mt*MetAP1c affects its enzyme activity**. (A) Deletion scheme. Notations used: WT, wild-type; Δ, deleted residues. (B) Assessment of methionine aminopeptidase activity of deletion variants. Methionine aminopeptidase activity assay was performed using indicated amount of wild-type and mutant proteins with 4 mM of Met-Ala-Ser as the substrate. (C) Coomassie stained SDS-PAGE gel (*upper panel*) and Western blot (*lower panel*) using anti- His antibody of the mutant proteins as shown in Figure 3B. (D) Far-UV CD spectra of the WT, Δ2-10 and Δ2-15 proteins. (E) Near-UV CD spectra of the WT, Δ2-10 and Δ2-15 proteins.

The presence of 40 amino acid N-terminal extension although not unusual among MetAP1s, its contribution towards the enzymatic activity is not known as yet [23-25]. To elucidate this aspect, a series of N-terminal deletion variants for *Mt*MetAP1c were generated (Figure 3A) and their enzyme activity was monitored (Figure 3B). Surprisingly, compared to the wild-type, deletion constructs (Δ2-10, Δ2-15, Δ2-20, Δ2-30 and Δ2-40) displayed either drastic decrease or no enzymatic activity (Figure 3B). Even use of 10 fold higher amounts of proteins in our assay, Δ2-10 or Δ2-15 exhibited only 30% activity while Δ2-20 or Δ2-30 or Δ2-40 had no activity (Figure 3B). Thus, our results argue that the catalytic domain alone is not sufficient for the enzymatic function of *Mt*MetAP1c.

*Mt*MetAP1c variants, like Δ2-20 or Δ2-30 or Δ2-40, which did not show any enzyme activity, when stained with Coomassie Brilliant Blue after resolving in SDS-PAGE, displayed a progressive loss in the purity of the proteins with the increase in the length of deleted regions (Figure 3C, *upper panel*). The authenticity of these mutant proteins was confirmed by Western blotting using anti-His antibody (Figure 3C, *lower panel*). The effect of mutation was evident by the presence of contaminating bands in the Ni-NTA purified samples (Figure 3C, *upper panel*) and even reduced protein yield (~4 mg for Δ2-20/Δ2-30/Δ2-40 as opposed to ~9 mg in wild-type average yield from 1 litre culture of *E. coli *cells). Analysis of its crystal structure revealed that the N-terminal extension present in *Mt*MetAP1c wraps around the surface of its catalytic domain in contiguous crevice [9]. Hence, its truncation as a result of deletion might have exposed the hydrophobic residues and thereby destabilized the protein. This has been manifested presumably by exhibiting altered proteolytic property of *Mt*MetAP1c variants and/or led to acquiring the tendency to associate with other proteins [9].

Interestingly, reduction in the enzymatic activity was also observed in Δ2-10 and Δ2-15 variants despite the fact that the Ni-NTA purified proteins appear to be similar in profile as has been observed with wild-type. The far-UV CD spectra showed that they (Δ2-10 and Δ2-15) are folded like the wild-type protein (Figure 3D). A slight alteration observed in the near-UV CD spectra for the Δ2-15 variant (Figure 3E) was very likely the result of alteration in the micro-environment of Trp present at the amino acid position 20. Nonetheless, it is apparent from our results that the first 15 amino acids of *Mt*MetAP1c are important for the catalytic activity of the enzyme.

### Role of Val-18 and Pro-19 on *Mt*MetAP1c enzyme activity

**Figure 4:**
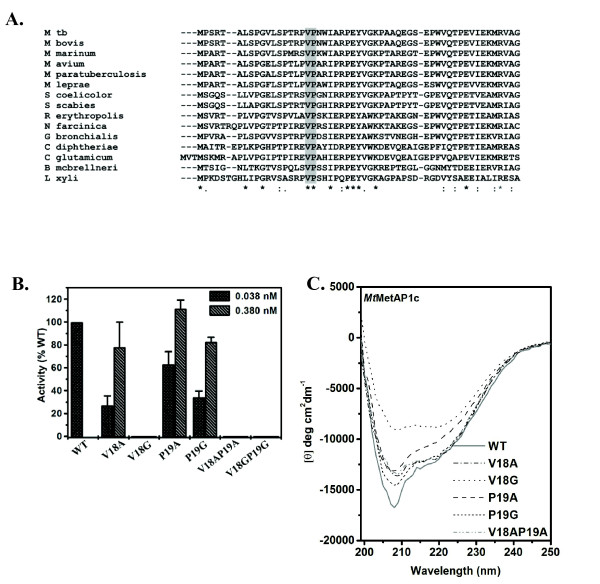
**Val-18 and Pro-19 at N-terminal extension are crucial for *Mt*MetAP1c enzyme activity**. (A) Sequence alignment of MetAP1c from different gram positive bacteria. Notations used with accession number in parantheses: M tb-*Mycobacterium tuberculosis *(P0A5J2); M bovis-*Mycobacterium bovis *(P0A5J3); M avium-*Mycobacterium avium *(A0QJ09); M marinum-*Mycobacterium marinum *(B2HJQ5); M paratuberculosis-*Mycobacterium paratuberculosis *(Q73VS7); M leprae-*Mycobacterium leprae *(Q9CBU7); S coelicolor-*Streptomyces coelicolor *(Q9RKR2); S scabies-*Streptomyces scabies *(C9ZHA6); R erythropolis-*Rhodococcus erythropolis *(C0ZY62); N farcinica-*Nocardia farcinica *(Q5YSA3); G bronchialis-*Gordonia bronchialis *(D0LBG0); C diphtheriae-*Corynebacterium diphtheriae *(Q6NGL5); C glutamicum-*Corynebacterium glutamicum *(Q6M437); B mcbrellneri-*Brevibacterium mcbrellneri *(D4YNZ0); L xyli-*Leifsonia xyli subsp. xyli *(Q6AFH6). (B) Effect of mutations on the *Mt*MetAP1c enzyme activity. The indicated amounts of wild-type and mutant proteins were used to monitor the enzyme activity with 4 mM Met-Ala-Ser as the substrate. (C) Far-UV CD spectra of the different mutant proteins.

To elucidate, the presence of any conserved residues between amino acid 15-20 of *Mt*MetAP1c, we aligned the amino acid sequence of the N-terminal extension region of the *Mt*MetAP proteins from Gram positive bacteria. Multiple sequence analysis revealed that Val-18 and Pro-19 are two highly conserved residues in different MetAPs (Figure 4A). To investigate their role, mutants replacing these two residues, one at a time (V18A, V18G, P19A, P19G) or both at the same time (V18AP19A and V18GP19G) were generated and the enzymatic activities of the recombinant proteins were monitored. Relative to wild type, V18A protein had ~30% activity while P19A and P19G exhibited ~65% and 35% activity respectively. On the other hand, V18G, V18AP19A and V18GP19G variants were devoid of any enzymatic activity (Figure 4B). Use of 10-fold excess of protein in assays, V18A, P19A and P19G mutants displayed activities almost at par (78%-112%) with the wild-type. However, use of V18G, V18AP19A and V18GP19G proteins even at increasing amounts in assays had hardly any activity (Figure 4B). These results indicated that Val-18 and/or Pro-19, the residues present in N-terminal extension (not in the catalytic domain) may contribute towards the enzymatic activity of *Mt*MetAP1c. Further, to monitor whether variation in the activity of the mutant proteins could be due to structural changes, far-UV CD studies were performed. The analysis of CD spectra revealed that mutations caused alteration in secondary structure of all the variants (Figure 4C). Compared to the wild type, mutation of Val-18 or Pro-19 (V18A, P19G, V18AP19A) resulted in the reduction of the negative band intensity in the region of 205-208 nm of the mutant proteins (Figure 4C). In the variant P19A, the reduction in band intensity was also observed at 222 nm (in addition to 205-208 nm), thereby indicating that the helical content was reduced. In V18G significant reduction was observed both at 205-208 nm and 222 nm. Thus, these results suggested the importance of these two residues (Val-18 and Pro-19) exclusively and mutually towards the enzymatic activity of *Mt*MetAP1c.

**Figure 5:**
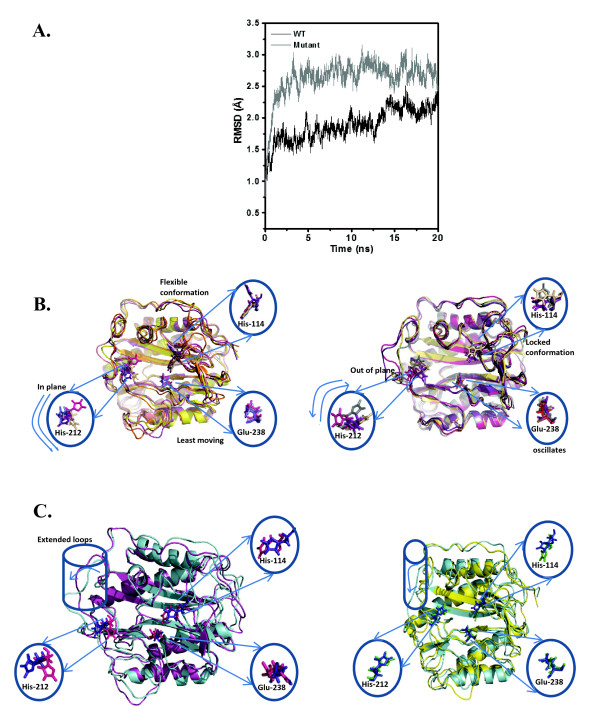
**Molecular dynamic simulations indicate Val-18 and Pro-19 maintain active site conformation of *Mt*MetAP1c**. (A) C_α _RMSD plot as a function of time at 300K over a period of 20 ns simulations done for wild-type and different mutant proteins. (B) MD simulations. The snapshots superimposed for wild-type (left) and V18GP19G (right) for 15 ns run. (*Inset*), depicts the highlighted regions of snapshots at 5ns, 7.5ns, 10ns, 15ns. (C) Snapshots of single mutant (left, V18G, blue-gray) superimposed with double mutant (pink). Both overlap each other to maximum probability compared to the wild-type protein. The loop proximal to the site of mutation is out of plane of the active site. MD simulations snapshots of single mutant (right, P19A, yellow ribbon blue sticks) superimposed with wild-type (blue-gray). Both overlap each other to maximum probability compared to the double mutant. The loop proximal to the site of mutation shows configurations similar to wild-type in 3-D space i.e. in plane to the active site.

To have an idea on how the Val-18 and Pro-19 of *Mt*MetAP1c influenced the activity of the enzyme, we used crystal structure of *Mt*MetAP1c to perform the Molecular dynamic (MD) simulation studies by introducing point mutation(s) at the site of interest (see 'Methods'). Four MD simulations were performed in explicit solvent conditions to examine the effect of specific mutations on overall structure and on the interactions of amino acids lining the active site of the protein. Comparison of the trajectories between wild-type and double mutant (V18GP19G) showed that overall fold of mutant protein is retained (Figure 5A). However, specific and drastic changes in the positions of active site residues were observed (Figures 5B and 5C). The conformational changes that initiated from the site of mutation (Val-18, Pro-19) propagated to the active site and displaced His-114 as well as affected His-212 and Glu-238. Analysis of trajectories of single mutants (V18G and P19A) illustrated that stereo chemical structural changes similar to that observed in double mutant (V18GP19G) were present in V18G (Figure 5C). On the contrary, MD simulation results of P19A appeared much similar to that of the wild-type but the presence of differential population of the ensemble at different time could possibly explain the reduction in the activity of this mutant protein (Figure 5C* right panel*). Thus, the results of our MD simulation studies support our biochemical observations.

## Discussion

MetAPs represent a unique class of metalloproteases that catalyze the co-translational removal of N-terminal methionine residue from the elongating polypeptide chain during the process of protein synthesis [2]. Since MetAP action in both prokaryotes and eukaryotes is mandatory during nascent protein synthesis, the importance of this enzyme has long been realized. Though the presence of two MetAP1s was known with the availability of the *M. tuberculosis *genome sequence, both of them (*Mt*MetAP1a and *Mt*MetAP1c) are active has been shown recently by others [17,19,20] and also in this study. However, it is still not known whether they are merely complementing each other for their functionality and therefore redundant within the genome or their presence is because of the specific needs of the bacterium. Recently, it has been reported that *Mt*MetAP1a knock down caused ~75% viability of mycobacteria while elimination of *Mt*MetAP1c resulted in 95% survival [20]. Since this enzyme from different sources has been considered as a potential drug target, identification of inhibitors against these enzymes, particularly for *M. tuberculosis Mt*MetAP1a is in progress [20]. However, the initial step of structure-activity relationship between these two proteins has not been addressed properly. In this context, to carry out systematic study of these two enzymes from *M. tuberculosis*, we have attempted here to analyze MetAP1s to elucidate subtle differences in their characteristics.

Our results indicated that *Mt*MetAP1c had strikingly high enzyme turnover rate with the same substrate (MAS) compared to *Mt*MetAP1a (~350-fold more; Table 1), although they had different substrate preferences (Figure 1A). The outcome of our CD as well as activity assays with these mycobacterial enzymes illustrate that *Mt*MetAP1a can sustain high temperature (up to 50°C) compared to *Mt*MetAP1c. In fact, T_m _value calculated based on CD spectra for *Mt*MetAP1a was 53.6°C as opposed to 42.7°C for *Mt*MetAP1c (Figure 1D). To get an insight into the cause of such differential behaviour of the two enzymes, structure of *Mt*MetAP1a was modelled and compared with the available structure of *Mt*MetAP1c (Figure 2B). We observed that the active site residues in both the *Mt*MetAP1s are essentially same, though their arrangement in space is different (Figure 2B). Furthermore, these amino acids are crucial because any alteration in them (for both the enzymes) yielded an inactive protein (Figure 2C). Thus it is logical to presume that this difference in the arrangement may be responsible for the alteration in their activity/functionality.

Sequence analysis revealed that the remarkable feature of *Mt*MetAP1c compared to *Mt*MetAP1a, is the presence of 40 amino acid long N-terminal extension. It has been suggested that this extension may be involved in the interaction of *Mt*MetAP1c with the ribosome [9]. In *Sc*MetAP1 and *Hs*MetAP2 deletion of N-terminal extension did not significantly alter the catalytic efficiency of the enzymes [23-25]. However, such deletion in *Sc*MetAP1 reduced its efficiency in rescuing the slow growth phenotype of a *map *mutant [26]. Interestingly, the zinc finger motif present in the N-terminal extension of *Sc*MetAP1 has been shown to be involved in its association with the ribosome [26]. In *Hs*MetAP1, on the other hand, these sequences have been implicated for the interaction of the enzyme with substrates and inhibitors [25]. This led us to explore the role of the N-terminal extension present in *Mt*MetAP1c towards the activity of the enzyme. We constructed a series of deletion mutants removing nine (Δ2-10), fourteen (Δ2-15), nineteen (Δ2-20), twenty-nine (Δ2-30) and thirty-nine (Δ2-40) amino acids from the amino terminal end of the *Mt*MetAP1c (Figure 3A). Enzyme assays with these constructs using even 10 fold excess of proteins compared to the wild-type revealed that there was a considerable loss in activity in Δ2-10 or Δ2-15 variants and no activity at all in mutants, like Δ2-20 or Δ2-30 or Δ2-40 (Figure 3B). Intriguingly, following resolving in SDS-PAGE, analysis of the deletion constructs in Coomassie Brilliant Blue stained gels reflected that purification profile of Δ2-10 or Δ2-15 mutants was similar to that of the wild-type but variants like Δ2-20, Δ2-30 and Δ2-40 exhibited loss in purity of the proteins (Figure 3C, *upper panel*). Hence, it seems logical to postulate that the amino acid residues between 15 and 20 of *Mt*MetAP1c are crucial for its activity. Perhaps, deletion of residues beyond 19 from the N-terminal end of *Mt*MetAP1c renders the enzyme unstable or exposes its hydrophobic patches, thereby increasing its tendency to associate with the other proteins. In fact, evaluation of structural changes through far-UV and near-UV CD spectra revealed that up to 15 residues from the N-terminal extension of *Mt*MetAP1c are dispensable for the folding of the protein (Figure 3D) but required for its activity (Figure 3B). Thus, our results argue that the N-terminal extension is important for the catalytic activity of *Mt*MetAP1c protein, which is unlike the reports available with *Sc*MetAP1, *Hs*MetAP1 and *Hs*MetAP2 [23-25].

To identify the critical residue(s) for the activity of the protein, we analyzed the sequence of MetAPs from different Gram positive bacteria with N-terminal extension and found the presence of conserved Val-18 and Pro-19 in between amino acid residues 15 and 20 (Figure 4A). This observation, prompted us to create point mutations at 18^th ^and 19^th ^position of *Mt*MetAP1c (one at a time or together with type-to-type/drastic substitutions) and examine their effect on the activity of the enzyme. While V18A and P19A were partially active, V18AP19A did not show any activity. On the other hand, P19G exhibited partial activity but neither V18G nor V18GP19G were active (Figure 4B). Further, to assess if these mutations resulted in any structural alterations, far-UV CD studies were carried out. Interestingly the mutation caused alteration in the secondary structure of the protein. Thus, our results argue the importance of these two residues (Val-18 and Pro-19) exclusively and mutually towards the enzymatic activity of *Mt*MetAP1c. It appears very likely that the structural alterations that occurred due to mutation at Val-18 and/or Pro-19 of *Mt*MetAP1c affected the active site conformation of the protein. In fact, MD simulations (both 5 ns and 50 ns) indicated that the conformational changes generated at the site of mutation (V18GP19G and V18G) propagated via connecting loops and helices to the enzyme active site resulting in the alteration in the movement/positioning of residues like His-114, His-212 and Glu-238 which are critical for the enzyme activity as revealed in our biochemical studies (Figure 2C). These variations in the V18GP19G or V18G mutant proteins presumably made the active site environment unfavourable for any activity. Thus, our results illustrate that residue(s) without being in the active site of an enzyme is capable of modulating its activity, which does not seem to be unusual [27,28].

## Conclusion

Our findings unequivocally emphasize that N-terminal extension of *Mt*MetAP1c contributes towards the functionality of the enzyme by regulating active site residues through "action-at-a-distance" mechanism and this is presumably its unique function in *Mt*MetAP1c, which we are reporting for the first time. Since Val-18 and Pro-19 are conserved residues throughout the Gram positive bacterial MetAP1s with N-terminal extension, it remains to be seen whether they are universal in contributing towards the functionality of the enzyme.

## Methods

### Materials

Restriction/modifying enzymes were obtained from New England Biolab. All other fine chemicals including Met-Gly, Met-Ala-Ser, Met-Gly-Met-Met, Gly-Gly-Ala were procured from Sigma Chemical Company. Ni-NTA resin (Qaigen), ECL Western blotting detection kit, PCR DNA/gel band purification kit, protein molecular weight markers (GE Healthcare) and Herculase fusion DNA polymerase (Stratagene) were commercially available. Oligonucleotides used in this study were custom synthesized (IDT/Ocimum Biosolutions/Sigma).

### DNA manipulation and generation of *Mt*MetAP-mutants

Genes encoding two MetAP1, *mapA *(Rv0734) and *mapB *(Rv2861c) of *M. tuberculosis *H37Rv (virulent strain) exhibited 100% identity at the nucleotide level with its avirulent strain (H37Ra). In this study, genomic DNA isolated [29] from *M. tuberculosis *strain H37Ra was used for PCR amplification of *mapA and mapB *genes. For this purpose, gene specific primers incorporating restriction sites were designed (primers CK74 with HindIII and CK140 with NheI sites for *mapA*; primers CK64 with NdeI and CK65 with HindIII sites for *mapB*; see Additional file 1, Table S1 for primer sequences) based on published genome sequence [30] and PCR reaction was carried out using Herculase fusion DNA polymerase following manufacturer's recommended protocol. This was followed by cloning of genes at NheI/HindIII (for *mapA*) or NdeI/HindIII (for *mapB*) sites in expression vector (pET-28c) using standard protocols [31] to obtain pET-mapA or pET-mapB construct. PCR was also employed for generation of different mutations (H88A, H193A, D117A, E219V and W236L for *Mt*MetAP1a and H114A, H212A, D131A, E238A, W255L, V18A, V18G, P19A, P19G, V18AP19A, V18GP19G for *Mt*MetAP1c) including deletion mutants (Δ2-10, Δ2-15, Δ2-16, Δ2-17, Δ2-18, Δ2-19, Δ2-20, Δ2-30, Δ2-40). For each point mutation two external (CK74/CK140 and CK64/CK65 for *mapA *and *mapB *respectively) and two internal primers (incorporating mutation) were designed (Additional file 1, Table S1). Two sets of primary (pET-mapA/pET-mapB as the template) and one set of secondary (mixture of primary reaction products as the template) PCR reactions were carried out for each mutation. For constructing deletion mutants, pET-mapB was used as template with two set of primers, one primer incorporating NdeI site (excluding N-terminal region to be deleted) and another was external primer (CK65). All these constructs including the wild-type (in pET-28c) were individually transformed into *E. coli *strains DH5α to build up DNA and in BL21 (DE3) for expression/purification of His-tagged proteins. All mutations were confirmed by sequencing using an automated DNA sequencer.

### Expression and purification of recombinant proteins

Cells harboring pET-mapA or pET-mapB or different mutant constructs were grown overnight (15 h at 37°C) in LB broth (50 μg/ml kanamycin) and induced with 0.4 mM IPTG at OD_600 _of ~0.8. Cells were harvested after 12 h following incubation at 16°C, resuspended in lysis buffer (100 mM Tris, pH 7.5 containing 300 mM NaCl for *Mt*MetAP1a or 200 mM NaCl for *Mt*MetAP1c, 10 mM imidazole, 1 mM phenylmethylsulfonyl fluoride, 1 μg/ml of pepstatin and 1 μg/ml of leupeptin or inhibitor cocktail, Roche) and sonicated. The supernatant fraction was further loaded on a Ni-NTA affinity column, washed with 10 bed volumes of 100 mM Tris pH 7.5 containing 300 mM (for *Mt*MetAP1a) or 200 mM (for *Mt*MetAP1c) NaCl with 20 mM imidazole. The purified protein(s) was eluted with elution buffer comprising of 100 mM Tris, pH 7.5 containing either 300 mM NaCl/250 mM imidazole (for *Mt*MetAP1a) or 200 mM NaCl/150mM imidazole (for *Mt*MetAP1c). Following removal of imidazole by dialysis at 4°C for ~14h (dialysis buffer: 100 mM Tris, pH 7.5 with either 300 mM NaCl for *Mt*MetAP1a or 150mM NaCl for *Mt*MetAP1c; buffer changed: 4 times), protein was estimated [32] and stored at -80°C until used.

### Methionine aminopeptidase activity

MetAP activity was measured by a colorimetric assay performed at 30°C in a microtiter plate by monitoring the absorbance of oxidized o-dianisidine at 440 nm [33]. Since the assay system involves the use of CoCl_2_, we determined its optimal amount (2 mM for *Mt*MetAP1a and 0.2 mM for *Mt*MetAP1c) to be used for the assessment of enzyme activity at our experimental conditions. We found use of 2 mM CoCl_2 _did not inhibit *Mt*MetAP1a enzyme activity and similar amount has also been used for *E. coli *MetAP enzyme assay [34]. Briefly, in a 100 μl reaction volume *Mt*MetAP1a (1.7 nM/reaction) or *Mt*MetAP1c (0.038 nM/reaction) protein was mixed with a reaction mixture consisting of 100 mM Tris-HCl pH 7.5, 0.2 mM (for *Mt*MetAP1c) or 2 mM (for *Mt*MetAP1a) CoCl_2_, 0.1mg/ml o-dianisidine, 3 units of horse radish peroxidase, 0.5 units of L-amino acid oxidase and 0.75-8 mM substrate (Met-Ala-Ser or Met-Gly-Met-Met). The activity of the enzyme was monitored in an ELISA plate reader for 2-15 min. The values obtained were corrected by subtracting the blank readings (no significant difference was noticed when assays were carried out with all ingredients except either substrate or protein) and μmol of product released was calculated by using the extinction coefficient [35] of oxidised o-dianisidine as 10580 M^-1^cm.^-1^.

### Western blotting

Purified proteins (1 μg protein/slot) were resolved in 12% SDS-PAGE and transferred to nitrocellulose membranes (0.45 μm) using a mini-transblot apparatus (Bio-Rad) for Western blotting. Membranes were stained with Ponceau S to ensure transfer, processed using anti-His/anti-mouse IgG and detected through ECL detection system following manufacturer's (GE Healthcare) recommended protocol.

### CD spectroscopy

CD spectra of *Mt*MetAP1a and *Mt*MetAP1c were recorded in a Jasco J-810 spectropolarimeter before and after thermal denaturation. Measurements in the far-ultraviolet region (190-250 nm) were performed on protein solutions (0.15-0.3 mg/ml in 20 mM Tris, pH 7.5 and 150 mM/300 mM NaCl) employing a cell with path length of 0.1 cm at 25°C. The protein samples were heated at a rate of 3°C/min up to 50°C and the spectra were recorded at 25°C and 50°C. For calculating T_m_, *Mt*MetAP1a and *Mt*MetAP1c were heated in the thermoelectric cell of the CD spectrophotometer at the fixed rate of 1°C/min and the thermal denaturation curves were recorded at 208 nm. Measurements in near-ultraviolet region (250-350 nm) were carried out using cell with path length of 1cm at 25°C on protein solutions (0.3-0.5 mg/ml in 20 mM Tris, pH 7.5 and 150 mM/300 mM NaCl). The mean residue ellipticity [θ] was calculated using a mean residue molecular mass of 110 Da. Each spectrum reported is an average of ten scans. Blank spectra of aqueous buffer were used to correct the observed spectra.

### Bioinformatic analysis

Nucleotide derived amino acid sequence of *Mt*MetAP1c was compared with non-redundant database using BLAST [36]. The multiple sequence alignment of the retrieved sequences was carried out using ClustalX [37] with default values for gap opening and extension penalties.

### Molecular modelling

The crystal structure of *Mt*MetAP1c (PDB ID: 1YJ3) [9] served as the template for modeling of *Mt*MetAP1a using Modeller 9v7 program and model was selected on the basis of DOPE score [38-40]. The PROCHECK validated model was energy minimised using LEap module of AMBER 9 package [41,42]. Structures of different mutants (V18G, P19G, and V18GP19G) were generated using *Mt*MetAP1c as the template and residue replacement editor in PYMOL. The structure of all the mutants were also energy minimized.

### Molecular dynamics simulations

All MD simulations were performed with AMBER 9.0 and FF03 forcefield [43]. The protein was embedded in the TIP3P water box [44], which approximately extended 10Å in each direction from the peripheral surface of the protein to any periodic box edge with unit box dimensions of 18.774 × 18.774 × 18.774 Å. Periodic boundary conditions were applied and the entire system (solvent with protein) was energy minimized. Initial equilibration was monitored for 1 ns and the production MD simulation was then run at 300K, maintained using Langevin weak coupling algorithm [45]. The simulation trajectory was calculated using heavy atom harmonic position SHAKE [46]. Simulation was continued till 50 ns with an integration step of 2 fs and snapshots were retained every 10 ps for further analysis. MD trajectories were analyzed using the PTRAJ module and structures were visualized using VMD molecular visualization program [47].

### Data analysis

Unless mentioned otherwise, reproducibility of each experiment throughout this study was checked at least three times and results were calculated as Mean ± SD.

## Abbreviations

anti-his: antibody against histidine-tag; IPTG: isopropyl-β-D-thiogalactopyranoside; *Hs*MetAP: *Homo sapiens *Methionine aminopeptidase; MD: molecular dynamic simulations; *Sc*MetAP1: *Saccharomyces cerevisiae *Methionine aminopeptidase 1

## Authors' contributions

PK and PKC conceived the study, planned the experiments, analyzed results and wrote the paper. PK did all wet lab experiments. MM and SK carried out Molecular modelling and Molecular dynamic simulation studies, interpreted results and wrote respective portions of the paper. All authors read and approved the final manuscript.

## Supplementary Material

Additional file 1 **Table S1**. Supplementary Table showing sequences of the primer used in this study. Click here for file

## References

[B1] Giglione C Boularot A Meinnel T Protein N-terminal methionine excision Cell Mol Life Sci 2004 61 12 1455 1474 15197470 10.1007/s00018-004-3466-8 PMC11138929

[B2] Giglione C Fieulaine S Meinnel T Cotranslational processing mechanisms: towards a dynamic 3D model Trends Biochem Sci 2009 34 8 417 426 10.1016/j.tibs.2009.04.003 19647435

[B3] Wiltschi B Merkel L Budisa N Fine tuning the N-terminal residue excision with methionine analogues Chembiochem 2009 10 2 217 220 10.1002/cbic.200800605 19067457

[B4] Solbiati J Chapman-Smith A Miller JL Miller CG Cronan JE Jr Processing of the N termini of nascent polypeptide chains requires deformylation prior to methionine removal J Mol Biol 1999 290 3 607 614 10.1006/jmbi.1999.2913 10395817

[B5] Meinnel T Mechulam Y Blanquet S Methionine as translation start signal: a review of the enzymes of the pathway in *Escherichia coli* Biochimie 1993 75 12 1061 1075 10.1016/0300-9084(93)90005-D 8199241

[B6] Arfin SM Kendall RL Hall L Weaver LH Stewart AE Matthews BW Bradshaw RA Eukaryotic methionyl aminopeptidases: two classes of cobalt-dependent enzymes Proc Natl Acad Sci USA 1995 92 17 7714 7718 10.1073/pnas.92.17.7714 7644482 PMC41216

[B7] Lowther WT Matthews BW Metalloaminopeptidases: common functional themes in disparate structural surroundings Chem Rev 2002 102 4581 4608 10.1021/cr0101757 12475202

[B8] Bradshaw RA Brickey WW Walker KW N-terminal processing: the methionine aminopeptidase and N alpha-acetyl transferase families Trends Biochem Sci 1998 23 7 263 267 10.1016/S0968-0004(98)01227-4 9697417

[B9] Addlagatta A Quillin ML Omotoso O Liu JO Matthews BW Identification of an SH3-binding motif in a new class of methionine aminopeptidases from *Mycobacterium tuberculosis *suggests a mode of interaction with the ribosome Biochemistry 2005 44 19 7166 7174 10.1021/bi0501176 15882055

[B10] Alvarado JJ Nemkal A Sauder JM Russell M Akiyoshi DE Shi W Almo SC Weiss LM Structure of a microsporidian methionine aminopeptidase type 2 complexed with fumagillin and TNP-470 Mol Biochem Parasitol 2009 168 2 158 167 10.1016/j.molbiopara.2009.07.008 19660503 PMC2759695

[B11] Hu X Addlagatta A Lu J Matthews BW Liu JO Elucidation of the function of type 1 human methionine aminopeptidase during cell cycle progression Proc Natl Acad Sci USA 2006 103 48 18148 18153 10.1073/pnas.0608389103 17114291 PMC1838721

[B12] Yeh JR Ju R Brdlik CM Zhang W Zhang Y Matyskiela ME Shotwell JD Crews CM Targeted gene disruption of methionine aminopeptidase 2 results in an embryonic gastrulation defect and endothelial cell growth arrest Proc Natl Acad Sci USA 2006 103 27 10379 10384 10.1073/pnas.0511313103 16790550 PMC1480595

[B13] Chen X Chong CR Shi L Yoshimoto T Sullivan DJ Jr Liu JO Inhibitors of *Plasmodium falciparum *methionine aminopeptidase 1b possess antimalarial activity Proc Natl Acad Sci USA 2006 103 39 14548 14553 10.1073/pnas.0604101103 16983082 PMC1599997

[B14] Chang SY McGary EC Chang S Methionine aminopeptidase gene of *Escherichia coli *is essential for cell growth J Bacteriol 1989 171 7 4071 4072 2544569 10.1128/jb.171.7.4071-4072.1989 PMC210164

[B15] Miller CG Kukral AM Miller JL Movva NR pepM is an essential gene in *Salmonella typhimurium* J Bacteriol 1989 171 9 5215 5217 2670909 10.1128/jb.171.9.5215-5217.1989 PMC210346

[B16] Li X Chang YH Amino-terminal protein processing in *Saccharomyces cerevisiae *is an essential function that requires two distinct methionine aminopeptidases Proc Natl Acad Sci USA 1995 92 26 12357 12361 10.1073/pnas.92.26.12357 8618900 PMC40356

[B17] Zhang X Chen S Hu Z Zhang L Wang H Expression and characterization of two functional methionine aminopeptidases from *Mycobacterium tuberculosis *H37Rv Curr Microbiol 2009 59 5 520 525 10.1007/s00284-009-9470-3 19688379

[B18] Chai SC Lu JP Ye QZ Determination of binding affinity of metal cofactor to the active site of methionine aminopeptidase based on quantitation of functional enzyme Anal Biochem 2009 395 2 263 264 10.1016/j.ab.2009.07.054 19712663 PMC2760622

[B19] Lu JP Chai SC Ye QZ Catalysis and inhibition of *Mycobacterium tuberculosis *methionine aminopeptidase J Med Chem 2010 53 3 1329 1337 10.1021/jm901624n 20038112 PMC2820511

[B20] Olaleye O Raghunand TR Bhat S He J Tyagi S Lamichhane G Gu P Zhou J Zhang Y Grosset J Bishai WR Liu JO Methionine aminopeptidases from *Mycobacterium tuberculosis *as novel antimycobacterial targets Chem Biol 2010 17 1 86 97 10.1016/j.chembiol.2009.12.014 20142044 PMC3165048

[B21] Lu JP Ye QZ Expression and characterization of *Mycobacterium tuberculosis *methionine aminopeptidase type 1a Bioorg Med Chem Lett 2010 20 9 2776 2779 10.1016/j.bmcl.2010.03.067 20363127 PMC2860377

[B22] Chiu CH Lee CZ Lin KS Tam MF Lin LY Amino acid residues involved in the functional integrity *of Escherichia coli *methionine aminopeptidase J Bacteriol 1999 181 15 4686 4689 10419973 10.1128/jb.181.15.4686-4689.1999 PMC103606

[B23] Zuo S Guo Q Ling C Chang YH Evidence that two zinc fingers in the methionine aminopeptidase from *Saccharomyces cerevisiae *are important for normal growth Mol Gen Genet 1995 246 2 247 253 10.1007/BF00294688 7862096

[B24] Yang G Kirkpatrick RB Ho T Zhang GF Liang PH Johanson KO Casper DJ Doyle ML Marino JP Jr Thompson SK Chen W Tew DG Meek TD Steady-state kinetic characterization of substrates and metal-ion speciicities of the full-length and N-terminally truncated recombinant human methionine aminopeptidases (type 2) Biochemistry 2001 40 10645 10654 10.1021/bi010806r 11524009

[B25] Li JY Chen LL Cui YM Luo QL Gu M Nan FJ Ye QZ Characterization of full length and truncated type I human methionine aminopeptidases expressed from *Escherichia coli* Biochemistry 2004 43 24 7892 7898 10.1021/bi0360859 15196033

[B26] Vetro JA Chang YH Yeast methionine aminopeptidase type 1 is ribosome-associated and requires its N-terminal zinc finger domain for normal function *in vivo* J Cell Biochem 2002 85 4 678 688 10.1002/jcb.10161 11968008

[B27] Ginsberg AM Spigelman M Challenges in tuberculosis drug research and development Nat Med 2007 13 3 290 294 10.1038/nm0307-290 17342142

[B28] Saxena R Kanudia P Datt M Dar HH Karthikeyan S Singh B Chakraborti PK Three consecutive arginines are important for the mycobacterial peptide deformylase enzyme activity J Biol Chem 2008 283 35 23754 23764 10.1074/jbc.M709672200 18574247 PMC3259783

[B29] Chaba R Raje M Chakraborti PK Evidence that a eukaryotic-type serine/threonine protein kinase from *Mycobacterium tuberculosis *regulates morphological changes associated with cell division Eur J Biochem 2002 269 4 1078 1085 10.1046/j.1432-1033.2002.02778.x 11856348

[B30] Cole ST Brosch R Parkhill J Garnier T Churcher C Harris D Gordon SV Eiglmeier K Gas S Barry CE Tekaia F Badcock K Basham D Brown D Chillingworth T Connor R Davies R Devlin K Feltwell T Gentles S Hamlin N Holroyd S Hornsby T Jagels K Krogh A McLean J Moule S Murphy L Oliver K Osborne J Deciphering the biology of *Mycobacterium tuberculosis *from the complete genome sequence Nature 1998 393 6685 537 544 10.1038/31159 9634230

[B31] Sambrook J Russel D Molecular Cloning: A Laboratory Manual 2001 3 Cold Spring Harbor Laboratory Press, Cold spring Harbor, NY

[B32] Bradford MM A rapid and sensitive method for the quantitation of microgram quantities of protein utilizing the principle of protein-dye binding Anal Biochem 1976 72 248 254 10.1016/0003-2697(76)90527-3 942051

[B33] Ben-Bassat A Bauer K Chang SY Myambo K Boosman A Chang S Processing of the initiation methionine from proteins: properties of the *Escherichia coli *methionine aminopeptidase and its gene structure J Bacteriol 1987 169 2 751 757 3027045 10.1128/jb.169.2.751-757.1987 PMC211843

[B34] Shapiro BA Gao N Thresher J Walkup KG Whiteaker J A high-throughput absorbance-based assay for Methionine produced by Methionine aminopeptidase using S-adenosyl-L-methionine synthetase J Biomol Screen 2011 in press 10.1177/1087057111398934 21402755

[B35] Frottin F Martinez A Peynot P Mitra S Holz RC Giglione C Meinnel T The proteomics of N-terminal methionine cleavage Mol Cell Proteomics 2006 5 12 2336 2349 10.1074/mcp.M600225-MCP200 16963780

[B36] Altschul SF Madden TL Schaffer AA Zhang J Zhang Z Miller W Lipman DJ Gapped BLAST and PSI-BLAST: a new generation of protein database search programs Nucleic Acids Res 1997 25 17 3389 3402 10.1093/nar/25.17.3389 9254694 PMC146917

[B37] Thompson JD Gibson TJ Plewniak F Jeanmougin F Higgins DG The CLUSTAL_X windows interface: flexible strategies for multiple sequence alignment aided by quality analysis tools Nucleic Acids Res 1997 25 24 4876 4882 10.1093/nar/25.24.4876 9396791 PMC147148

[B38] Sali A Blundell TL Comparative protein modeling by satisfaction of spatial restraints J Mol Biol 1993 234 779 815 10.1006/jmbi.1993.1626 8254673

[B39] Shen MY Sali A Statistical potential for assessment and prediction of protein structures Protein Sci 2006 15 11 2507 2524 10.1110/ps.062416606 17075131 PMC2242414

[B40] Eramian D Shen MY Devos D Melo F Sali A Marti-Renom MA A composite score for predicting errors in protein structure models Protein Sci 2006 15 7 1653 1666 10.1110/ps.062095806 16751606 PMC2242555

[B41] Laskowski RA MacArthur MW Moss DS Thornton JM PROCHECK: a program to check the stereo chemical quality of protein structures J Appl Cryst 1993 26 283 291 10.1107/S0021889892009944

[B42] Case TADDA Cheatham TE III Simmerling CL Wang J Duke RE Luo KMMR Pearlman DA Crowley M Walker RC Zhang W Wang B Hayik ARS Seabra KF Wong F Paesani X Wu S Brozell V Tsui H Gohlke LYC Tan J Mongan V Hornak G Cui P Beroza DH Mathews C Schafmeister WSR Kollman PA AMBER 9 2006 University of California, SanFrancisco

[B43] Duan Y Wu C Chowdhury S Lee MC Xiong G Zhang W Yang R Cieplak P Luo R Lee T Caldwell J Wang J Kollman P A point-charge force field for molecular mechanics simulations of proteins based on condensed-phase quantum mechanical calculations J Comput Chem 2003 24 16 1999 2012 10.1002/jcc.10349 14531054

[B44] Price DJ Brooks CL A modified TIP3P water potential for simulation with Ewald summation J Chem Phys 2004 121 10096 10103 10.1063/1.1808117 15549884

[B45] Futaba DN Hata K Yamada T Hiraoka T Hayamizu Y Kakudate Y Tanaike O Hatori H Yumura M Iijima S Shape-engineerable and highly densely packed single-walled carbon nanotubes and their application as super-capacitor electrodes Nat Mater 2006 5 12 987 994 10.1038/nmat1782 17128258

[B46] Ryckaert JP Ciccotti Berendsen HJC Numerical integration of the Cartesian equations of motion of a system with constraints: molecular dynamics of n-alkanes J Comput Phys 1977 23 327 341 10.1016/0021-9991(77)90098-5

[B47] Humphrey W Dalke A Schulten K VMD: visual molecular dynamics J Mol Graph 1996 14 1 33 38 27-38 10.1016/0263-7855(96)00018-5 8744570

