# Supplementary material for: Amino-terminal extension present in the methionine aminopeptidase type 1c of Mycobacterium tuberculosis is indispensible for its activity
Source: BMC Biochem. 2011 Jul 5;12:35. doi: 10.1186/1471-2091-12-35 (PMC3154147; doi:10.1186/1471-2091-12-35)
Supplement: Additional file 1 — Table S1. Supplementary Table showing sequences of the primer used in this study. [file 1471-2091-12-35-S1.DOC]

**Additional file**

**Table S1.** **List of primers used in this study**

Primer Sequence

CK 74 (*mapA* antisense) 5’ ATCTGCCCAAGCTTCTAACCGAGCGTCAGAATT 3’

CK 140 (*mapA* sense) 5’ AACTATGCTAGCATGCGCCCACTGG 3’

CK 64 (*mapB* sense) 5’ atactatctgggatcccatatgcctagtcgtaccg 3’

CK 65 (*mapB* antisense) 5’ tactatcaagcttctacagacaggtcaggatctcgacg 3’

CK 79(H114A sense) 5’ GAGGTCATCTGCGCTGGAATCCCCGAC 3’

CK 80(H114A antisense) 5’ GTCGGGGATTCCAGCGCAGATGACCTC 3’

CK 83(H212A sense) 5’ GCACGACGTTCGCCAACGGGCTGGTC 3’

CK 84(H212A antisense) 5’ GACCAGCCCGTTGGCGAACGTCGTGC 3’

CK 109 (D131A sense) 5’ ATCGTCAACATCGCCGTCACCGCCTA 3’

CK 110 (D131A antisense) 5’ TAGGCGGTGACGGCGATGTTGACGAT 3’

CK 87(E238A sense) 5’ ACCTTCACCATCGCGCCGATGATCAAC 3’

CK 88(E238A antisense) 5’ GTTGATCATCGGCGCGATGGTGAAGGT 3’

CK 167 (W255L sense) 5’ TGGGACGACGGTTTGACGGTGGTCACC 3’

CK 168 (W255L antisense) 5’ GGTGACCACCGTCAAACCGTCGTCCCA 3’

CK 161 (H88A sense) 5’ GACCGTGTGGTTGCTGGCATCCCGTCG 3’

CK 162 (H88A antisense) 5’CGACGGGATGCCAGCAACCACACGGTC 3’

CK 234 (H193A sense) 5’ GGACGACAGATGGCTATGGATCCGTTCTTG 3’

CK 235 (H193A antisense) 5’ CAAGAACGGATCCATAGCCATCTGTCGTCC 3’

CK 147 (D117A sense) 5’ GGTTGGCATGGCGCTGCGGCGATCACT 3’

CK 148 (D117A antisense) 5’ AGTGATCGCCGCAGCGCCATGCCAACC 3’

CK 151 (E219V sense) 5’ GTGCTGGCCATCGTACCGATGCTGACC 3’

CK 152 (E219V antisense) 5’ GGTCAGCATCGGTACGATGGCCAGCAC 3’

CK 159 (W236L sense) 5’ CTCGACGACAAACTGACGGTCACGACC 3’

CK 160 (W236L antisense) 5’ GGTCGTGACCGTCAGTTTGTCGTCGAG 3’

**N-terminal deletion primers**

CK183 (Δ2-10 sense) 5’ CATACTAGGATCCCATATGGTGCTGTCCCCGACA 3’

CK 66 (Δ2-15 sense) 5’ CATACTATGGATCCCATATGCGGCCGGTGCCC 3’

CK 217 (Δ2-16 sense) 5’ CATACTATGGATCCCATATGCCGGTGCCCAACTGGATC 3’

CK 218 (Δ2-17 sense) 5’ AATATTATGGATCCCATATGGTGCCCAACTGGA 3’

CK 219 (Δ2-18 sense) 5’ AATATTATGGATCCCATATGCCCAACTGGATTGCG 3’

CK 221 (Δ2-19 sense) 5’ TATTATCATATGAACTGGATTGCGCGC 3’

CK184 (Δ2-20 sense) 5’ CATACTACATATGTGGATCGCGCGC 3’

CK185 (Δ2-30 sense) 5’ CATACTAGGATCCCATATGCCGGCCGCCCAAGA 3’

CK133 (Δ2-40 sense) 5’ ACTATCTGGGATCCCATATGGTGCAGACACCTGAG 3’

**Primers for generating point mutants of N-terminal residues**

CK222 (V18A sense) 5’ TCCCCCACACGGCCGGCGCCCAACTGGATC 3’

CK223 (V18A antisense) 5’ GATCCAGTTGGGCGCCGGCCGTGTGGGGGA 3’

CK224 (V18G sense) 5’ CCAACACGGCCGGGACCCAACTGGATC 3’

CK225 (V18G antisense) 5’ GATCCAGTTGGGTCCCGGCCGTGTTGG 3’

CK226 (P19A sense) 5’ ACACGGCCGGTGGCAAACTGGATCGCG 3’

CK227 (P19A antisense) 5’ CGCGATCCAGTTTGCCACCGGCCGTGT 3’

CK228 (P19G sense) 5’ACACGGCCGGTGGGCAACTGGATCGCG 3’

CK229 (P19G antisense) 5’ CGCGATCCAGTTGCCCACCGGCCGTGT 3’

CK230 (V18AP19A sense) 5’CCCACACGGCCGGCAGCAAACTGGATCGCGCGC 3’

CK231 (V18AP19A antisense) 5’ GCGCGCGATCCAGTTTGCTGCCGGCCGTGTGGG 3’

CK232 (V18GP19G sense) 5’ CCAACACGGCCGGGAGGAAACTGGATCGCGCGC 3’

CK233 (V18GP19G antisense) 5’GCGCGCGATCCAGTTTCCTCCCGGCCGTGTTGG 3’
